# Supplementary material for: Delivery of monoclonal antibodies using mRNA lipid nanoparticles confers protection against SARS-CoV-2 and influenza
Source: Mol Ther Nucleic Acids. 2026 Feb 26;37(2):102873. doi: 10.1016/j.omtn.2026.102873 (PMC12996773; doi:10.1016/j.omtn.2026.102873)
Supplement: Document S1. Figures S1–S3 and Tables S1 and S2 [file mmc1.pdf]

OMTN, Volume *37*

Supplemental information

Delivery of monoclonal antibodies using mRNA lipid nanoparticles confers protection against SARS-CoV-2 and influenza

Mai N. Vu, Jessica A. Neil, Charley Mackenzie-Kludas, Andrew Kelly, Hyon-Xhi Tan, Kanta Subbarao, Wen Shi Lee, and Adam K. Wheatley

Table S1: Formulation of mAb mRNA/LNPs

Formulation	Lipid components*	Lipid ratio*	N:P ratio
ALC-0315	ALC-0315, DSPC, cholesterol, ALC-0159	46.3/9.4/42.7/1.6	1:5
MC3/DOTAP	Dlin-MC3-DMA, DSPC, cholesterol, DSPE-PEG2000, DOTAP	25.0/5.0/19.2/0.8/50.0	1:6

*Ionizable lipid, helper lipid, cholesterol, PEG-lipid, cationic lipid

Table S2: Characterisation of mRNA/LNPs

Formulation	mRNA	Diameter (nm)	PdI	Zeta potential (mV)	Encapsulation efficiency (%)
ALC-0315	HC/LC admix	62.3 ± 1.5	0.102	- 8.31	89.3
	HC-P2A-LC	64.1 ± 1.3	0.116	- 8.09	90.6
MC3-DOTAP	HC-P2A-LC	121.7 ± 4.2	0.146	+ 15.2	96.5

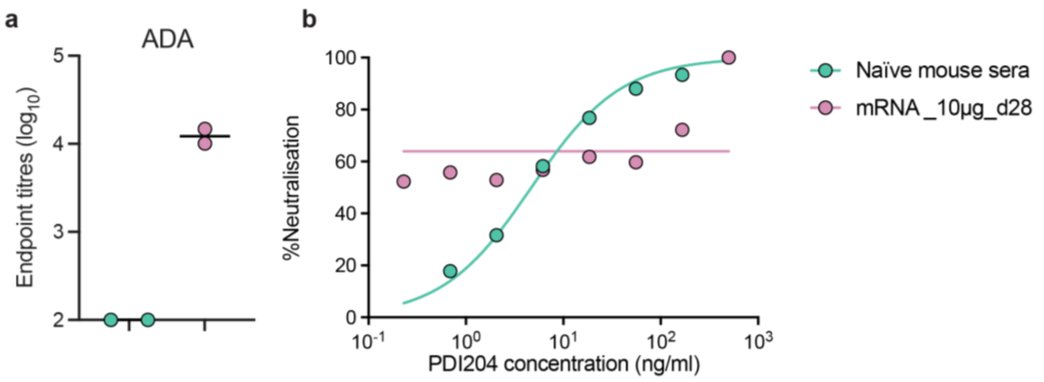

Figure S1: Effects of anti-PDI204 ADA on neutralisation activity of PDI204. Sera from naïve mice and from mice intravenously injected with 10 µg PDI204 mRNA/LNPs were collected at day 28 (the latest time point at which PDI204 was detectable) and pooled. (a) Anti-PDI204 ADA titres in pooled sera. (b) Neutralisation activity of PDI204 in the presence of pooled mouse sera against ancestral SARS-CoV-2 measured by a microneutralisation assay.

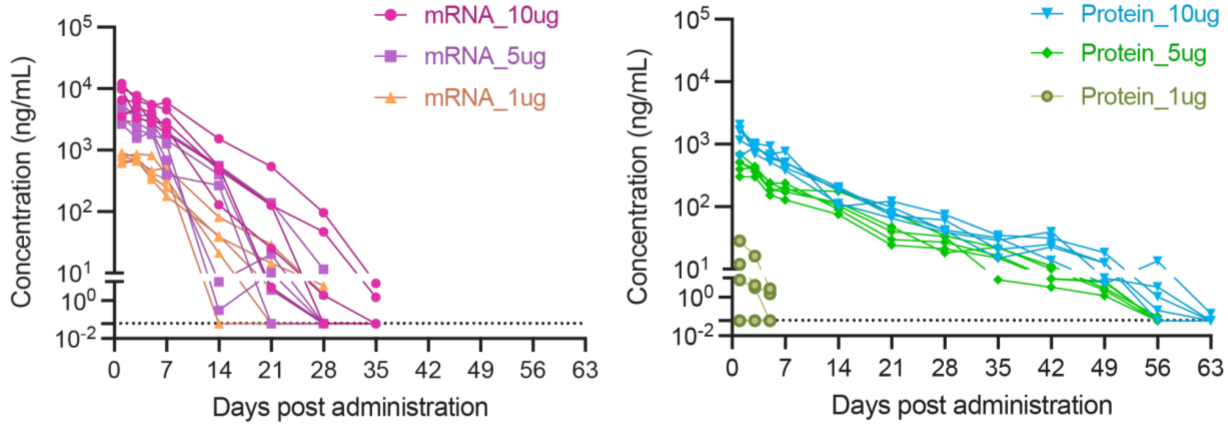

Figure S2: **PK profile of individual animals administered with HV-B10 mRNA/LNPs and protein.** HV-B10 mRNA/LNPs or proteins at 1, 5, or 10 µg were i.v. injected into C57BL/6 mice (n = 5). Sera from injected mice were collected at 1, 3, 5, 7, 14, 21, 28, 35, 42, 49, 56, and 63 days post injection. HV-B10 concentrations in the sera over time were calculated using ELISA against A/California/04/2009 HA protein (related to Figure 4b).

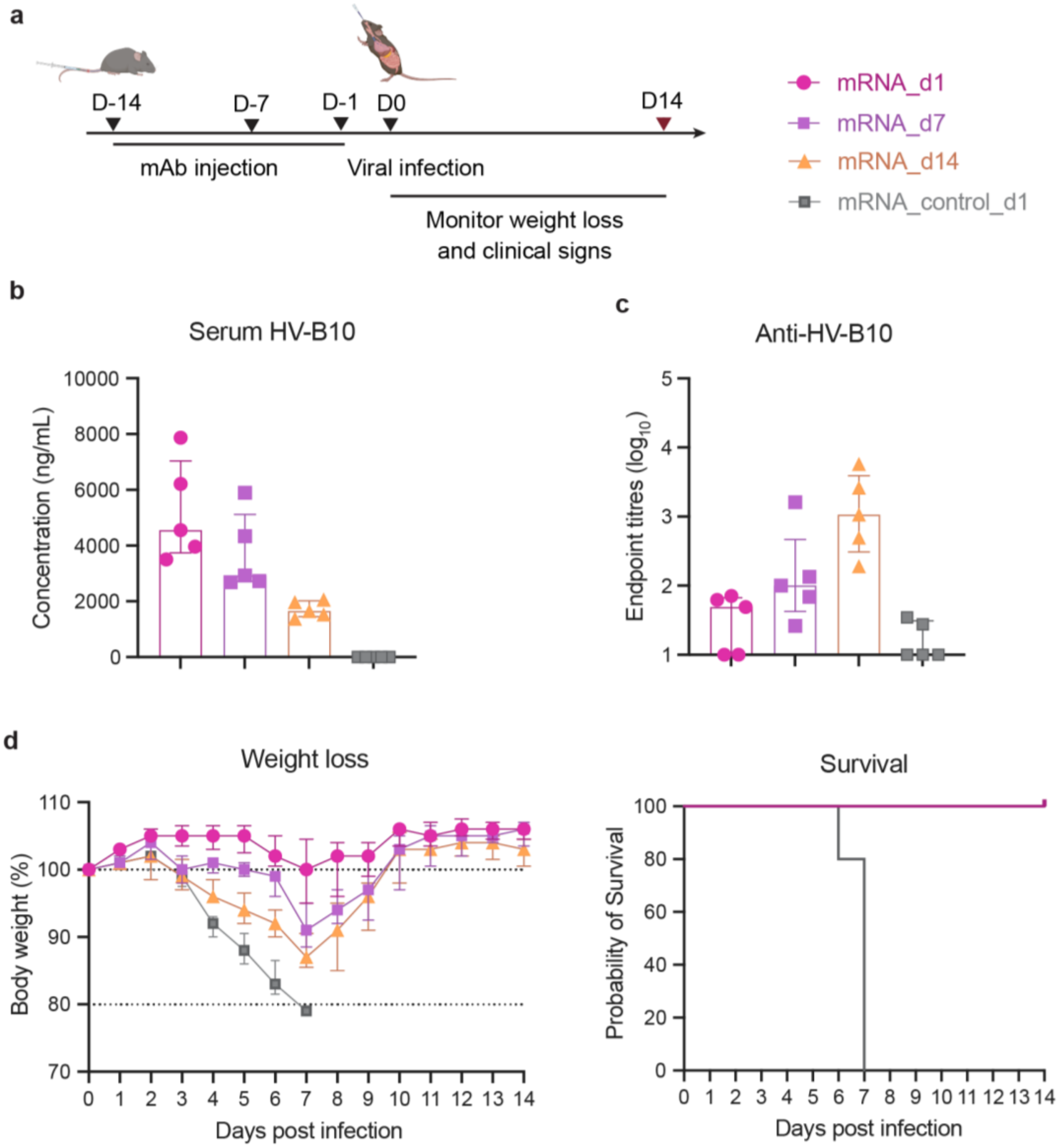

Figure S3: Timing of administration affects the protection efficacy of HV-B10 mRNA/LNPs against influenza infection. a). HV-B10 mRNA/LNPs at 5 μg were i.v. injected into C57BL/6 mice (n = 5) at day -14, -7, and -1 and an irrelevant mAb mRNA/LNPs at 5 μg were i.v. injected at day -1 as a negative control. At day 0, the mice were i.n. infected with 100 pfu of A/California/04/2009 virus. b). HV-B10 concentrations in the sera collected prior to infection were calculated using ELISA against A/California/04/2009 HA protein. c). Anti-HV-B10 anti-drug antibody titres in the mouse serum were measured by an ELISA assay. d). Changes in body weight (left) and survival rates (right) of the mice in 14 days post viral challenge. Data are shown as median ± IQR.
