# Supplementary material for: Delivery of monoclonal antibodies using mRNA lipid nanoparticles confers protection against SARS-CoV-2 and influenza
Source: Mol Ther Nucleic Acids. 2026 Feb 26;37(2):102873. doi: 10.1016/j.omtn.2026.102873 (PMC12996773; doi:10.1016/j.omtn.2026.102873)
Supplement: Document S2. Article plus supplemental information [file mmc2.pdf]

Molecular Therapy
Nucleic Acids
Original Article

Delivery of monoclonal antibodies using mRNA lipid nanoparticles confers protection against SARS-CoV-2 and influenza

Mai N. Vu,[1] Jessica A. Neil,[1] Charley Mackenzie-Kludas,[1] Andrew Kelly,[1] Hyon-Xhi Tan,[1] Kanta Subbarao,[1] Wen Shi Lee,[1] and Adam K. Wheatley[1]

[1]Department of Microbiology and Immunology, The University of Melbourne, at The Peter Doherty Institute for Infection and Immunity, Melbourne, VIC 3000, Australia

Monoclonal antibodies (mAbs) are an emerging class of therapeutics for the prevention and treatment of viral infections. Recent advances in mRNA/lipid nanoparticle (LNP) technology provide a promising new modality for the production of mAbs *in vivo*, potentially bypassing the need for recombinant manufacturing of mAb proteins. In this study, we compared traditional infusion of protein-based neutralizing mAbs targeting severe acute respiratory syndrome coronavirus 2 (SARS-CoV-2) or influenza viruses to mRNA/LNP-based production of mAbs in treated mice. High serum concentrations of mAbs were achieved upon delivery of a single mRNA encoding both heavy and light chains via intravenous or intramuscular routes using prototypic LNP formulations. However, the pharmacokinetics of mRNA-delivered mAbs were heavily influenced by the induction of anti-drug antibody responses directed against the encoded mAbs, resulting in reduced half-life *in vivo* and compromised protective capacity against SARS-CoV-2 Omicron BA.1 infection. In contrast, mRNA/LNP delivery of a neutralizing mAb conferred superior protection against lethal influenza challenge compared to equivalent recombinant protein doses. Overall, mRNA/LNP delivery comprises a feasible and attractive pathway to speed the development and deployment of antiviral antibodies. However, optimization of LNP formulation, dosing, and administration routes is required to maximize protective potential.

INTRODUCTION

Viral pathogens constitute a persistent threat to global health and economic security. Neutralizing antibodies, which can directly block infection, are a key correlate of immune protection.[1,2] Highly potent neutralizing monoclonal antibodies (mAbs) have been developed as effective antiviral drugs for several viral diseases, such as Ebola,[3] respiratory syncytial virus,[4] and severe acute respiratory syndrome coronavirus 2 (SARS-CoV-2).[5] However, for endemic viruses, the high production costs historically associated with protein mAb-based treatments have been a barrier to development, while in the context of outbreaks or pandemics, the long lead times required for cell-based recombinant manufacture significantly delay the potential introduction of mAbs to protect vulnerable populations.

Technological advances in the delivery of mRNA encapsulated within lipid nanoparticles (LNPs) have transformed vaccine delivery, with products from Pfizer/BioNTech[6] and Moderna[7] demonstrating considerable clinical impact during the recent coronavirus disease 2019 (COVID-19) pandemic. Adapting mRNA-LNP technology to bypass the complex cell-based manufacturing of traditional recombinant mAbs, including the purification process and post-translation modification concerns, could provide a cost-effective, flexible, and efficient method to deliver mAb therapeutics.[8] However, the potential for mRNA/LNPs in the delivery of antibody-based drugs is relatively understudied, with optimal parameters for mRNA design, LNP formulation, and route of administration yet to be comprehensively defined. Intravenous (i.v.) administration of LNPs co-encapsulating two separate mRNAs encoding the heavy (HC) and light immunoglobulin chains (LC) has achieved marked serum concentration of mAbs against SARS-CoV-2[9] or severe fever with thrombocytopenia syndrome virus (SFTSV)[10] in pre-clinical models. In a phase I clinical trial, mRNA/LNP delivery of a mAb targeting Chikungunya virus was safe in humans and similarly induced robust serum titers.[11] However, optimizing LNP formulations, mRNA formats, and administration routes for efficient delivery of mRNA-encoded mAbs to both systemic and mucosal compartments requires further exploration. This is particularly critical in the treatment of respiratory viruses, where mucosal localization of mAbs may be crucial to prevent viral entry, replication, and transmission.[12,13]

Received 22 August 2025; accepted 23 February 2026;
https://doi.org/10.1016/j.omtn.2026.102873.

Correspondence: Wen Shi Lee, Department of Microbiology and Immunology, The University of Melbourne, at The Peter Doherty Institute for Infection and Immunity, Melbourne, VIC 3000, Australia.
E-mail: ws.lee@unimelb.edu.au
Correspondence: Adam K. Wheatley, Department of Microbiology and Immunology, The University of Melbourne, at The Peter Doherty Institute for Infection and Immunity, Melbourne, VIC 3000, Australia.
E-mail: a.wheatley@unimelb.edu.au

Published by Elsevier Inc. on behalf of The American Society of Gene and Cell Therapy.
This is an open access article under the CC BY license (http://creativecommons.org/licenses/by/4.0/).

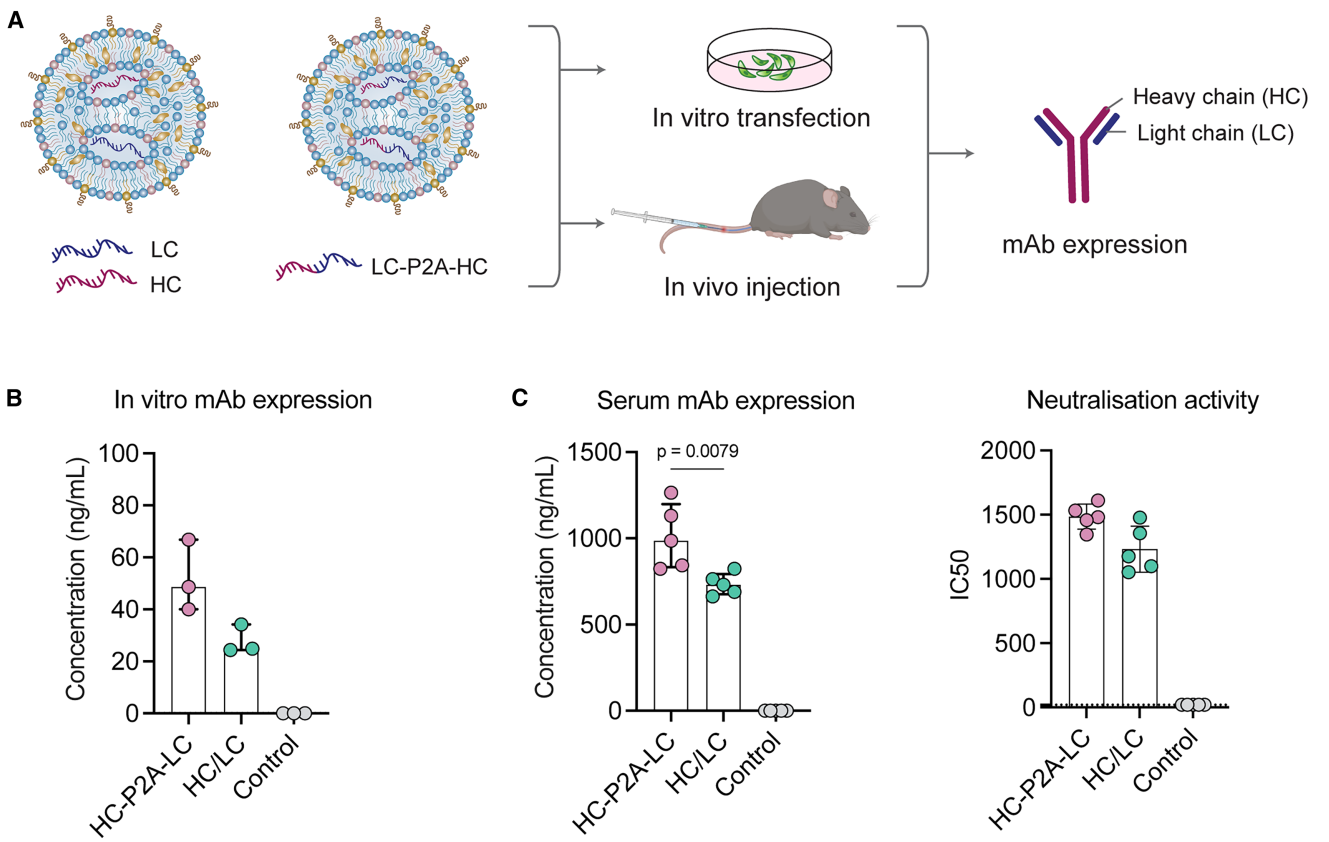

Figure 1. Rational design and characterization of mRNA/LNPs delivering monoclonal antibodies

(A) LNPs delivering PDI204 HC and LC encoded in two separate mRNAs or in a single mRNA with a P2A linker were transfected into HEK293T cells or i.v. injected into C57BL/6 mice (n = 5 per group). LNPs carrying ovalbumin mRNA were used as a negative control. (B) Concentrations of PDI204 expression in HEK293T cells at 48 h after transfection, measured by ELISA against the SARS-CoV-2 spike protein. (C) Concentrations of PDI204 expression in mouse serum at 48 h post-injection, measured by ELISA against SARS-CoV-2 spike protein (left), and neutralization activities of the mouse sera against ancestral SARS-CoV-2 virus using a microneutralization assay (right). Data are shown as median ± IQR. Statistical significance was determined using a Kruskal-Wallis test followed by post-hoc Dunn's multiple comparisons test.

Here, we contrasted mRNA/LNP versus conventional delivery of two potent neutralizing human IgG1 mAbs against COVID-19 (PDI204) and influenza (HV-B10) viruses.[14,15] A single mRNA construct encoding both HC and LC drove robust mAb expression in mice and readily detectable serum neutralizing titers. Classical LNP formulations analogous to the Pfizer/BioNTech COVID-19 vaccine (Comirnaty) elicited high mAb titers within the blood and lung after i.v. or intramuscular (i.m.) administration but not following intranasal (i.n.) instillation. In comparison to recombinant protein controls, mRNA-delivered mAbs elicited comparable peak serum concentrations but were subject to more rapid waning, likely attributable to the induction of anti-drug antibodies (ADA), heightened by the adjuvant properties of LNP formulations. Finally, in experimental challenge models of influenza or SARS-CoV-2, we found that mRNA/LNP delivery of mAbs could provide a degree of protection against the development of severe disease, although protective efficacy was modulated by dose, pathogen, and timing of challenge. Our study highlights that mRNA/LNPs constitute a tractable pathway for the rapid and efficient delivery of protective antibodies against viral diseases.

RESULTS

Expression of functional mAb *in vitro* and *in vivo* following mRNA/LNP delivery

Previously, we isolated a potent SARS-CoV-2 neutralizing human IgG1 mAb, PDI204, which targets the receptor-binding domain (RBD) of the spike protein.[14] The safety and efficacy of this mAb in humans are being evaluated in a phase I clinical trial (NCT06965751). Here, we explored the potential of mRNA/LNPs to deliver PDI204 using two mRNA formats: (i) encoding the HC and LC separately in two mRNA constructs or (ii) combining the two chains in a single mRNA construct linked by a furin cleavage site and a self-cleaving peptide P2A motif[16] (HC-P2A-LC). Using a formulation analogous to Comirnaty (ALC-0315) (Table S1),[17] we prepared LNPs carrying an admix of HC and LC mRNAs (at a 1:1 molar ratio) or HC-P2A-LC mRNA for *in vitro* and *in vivo* delivery (Figure 1A). Similar physicochemical properties (diameters

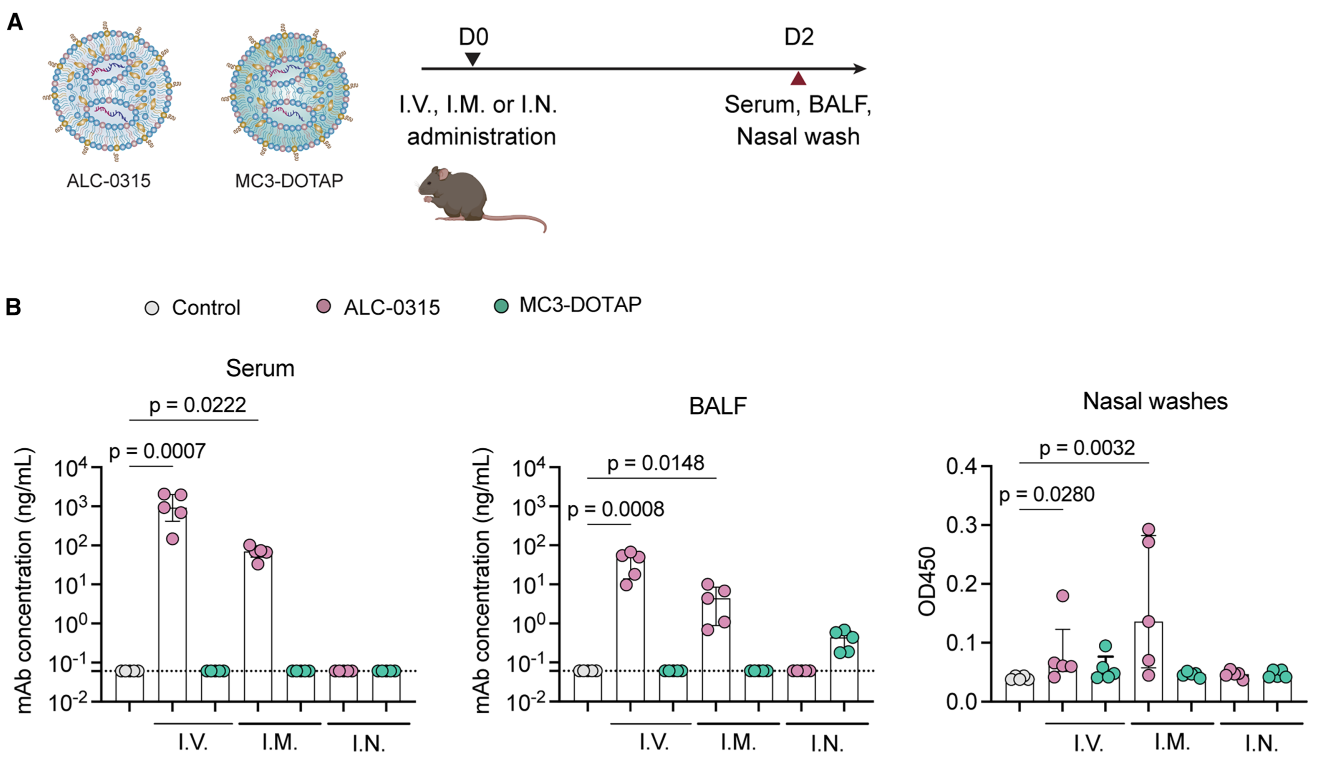

Figure 2. Effects of LNP formulations and administration routes on biodistribution of mRNA-delivered mAbs *in vivo*

(A) ALC-0315 and MC3-DOTAP LNPs delivering PDI204 HC-P2A-LC mRNAs at 5 μg were injected into C57BL/6 mice (n = 5) via i.v., i.m., or i.n. routes. At 48 h post-injection, serum, BALF, and nasal wash samples from the injected mice were collected. (B) Levels of PDI204 in serum and mucosal washes were measured by ELISA against SARS-CoV-2 spike protein. Data are shown as median ± IQR. Statistical significance was determined using a Kruskal-Wallis test followed by post-hoc Dunn's multiple comparisons test.

of ∼60 nm, negative charges of approximately – 8 mV, and encapsulation efficiencies of ∼90%) were observed between these two LNPs (Table S2).

The expression of functional PDI204 mAbs following mRNA/LNP transfection of HEK293T cells was confirmed by ELISA, with no marked differences in the concentrations of mAbs expressed in supernatants between the two mRNA formats (Figure 1B). However, at 48 h after i.v. administration into C57BL/6 mice, the combined HC-P2A-LC mRNA/LNPs produced significantly higher serum PDI204 concentrations (p = 0.0079) and greater neutralization activity compared to delivery of the admixed HC/LC mRNAs (Figure 1C). Overall, both delivery approaches demonstrated the capacity to efficiently deliver functional PDI204 mAbs in cell culture and mice, with the combined HC-P2A-LC mRNA construct selected for the following experiments.

Effects of LNP formulation and administration routes on the biodistribution of mRNA-delivered mAbs

Positively charged LNP formulations have been reported to enhance mRNA delivery to the lungs following i.v. injection;[18,19] however, our previous studies have highlighted that both anionic and cationic LNPs can efficiently deliver mRNA to the respiratory mucosa following i.n. administration.[17] Here, to assess the effects of LNP formulation and delivery routes on mAb biodistribution, we utilized these two LNP formulations developed previously[17] to encapsulate PDI204 HC-P2A-LC mRNA: (i) an anionic, Comirnaty-like ALC-0315 LNP, and (ii) a cationic MC3-DOTAP LNP (Table S1). Compared to ALC-0315 (∼64.1 nm, −8.09 mV), the MC3-DOTAP LNPs had a larger diameter of ∼121.7 nm and a positive charge of +15.2 mV (Table S2).

PDI204 ALC-0315 and MC3-DOTAP mRNA/LNPs were delivered into mice via i.v., i.n., and i.m. injection. PDI204 expression in mouse serum and mucosal fluids, including bronchoalveolar lavage fluid (BALF) and nasal washes,[17] was quantified at 48 h post-administration (Figure 2A). i.v. and i.m. delivery of ALC-0315 LNPs drove the accumulation of higher levels of PDI204 in serum, BALF, and nasal wash samples compared to animals treated with ovalbumin control mRNA/LNPs (Figure 2B), but minimal expression was detected at any site following i.n. delivery. In contrast, MC3-DOTAP LNPs were poor at driving mAb expression *in vivo*, although there was evidence of some expression within the lungs following i.n. administration. Overall, ALC-0315-based formulations and i.v. and i.m. routes appeared best suited to deliver mAbs *in vivo*.

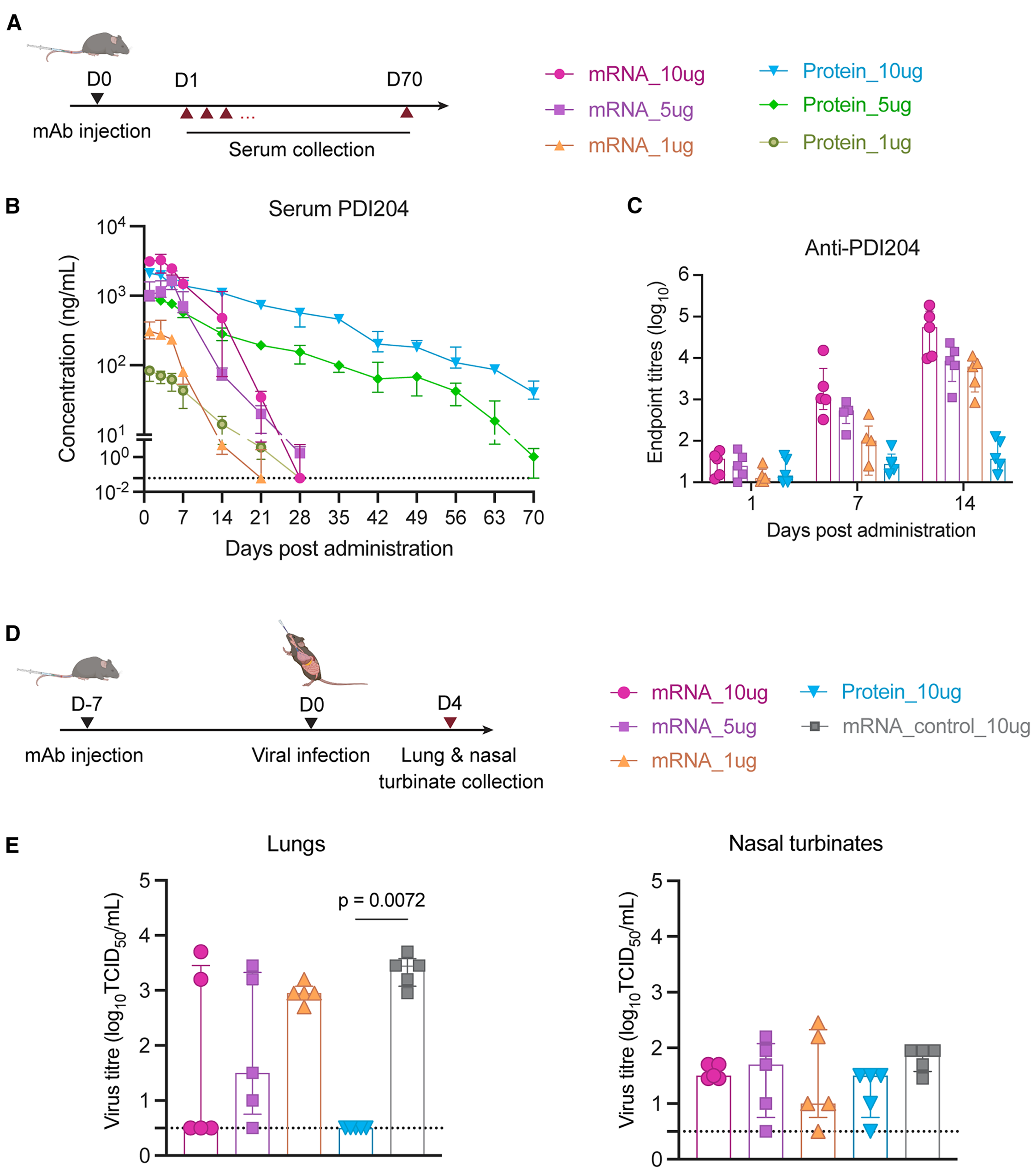

Figure 3. PK profile and prophylactic efficacy of PDI204 mRNA/LNPs against SARS-CoV-2 infection

(A) PDI204 mRNA/LNPs or proteins at 1, 5, or 10 μg were i.v. injected into C57BL/6 mice (*n* = 5). Sera from injected mice were collected at 1, 3, 5, 7, 14, 21, 28, 35, 42, 49, 56, 63, and 70 days post-injection. (B) PDI204 concentrations in sera over time were calculated using ELISA against SARS-CoV-2 spike protein. (C) Anti-PDI204 antibody titers in mouse serum at days 1, 7, and 14 post-injection were measured by ELISA assay. (D) k18-hACE2 mice (*n* = 5) were i.v. injected with PDI204 mRNA/LNPs at 1, 5, or 10 μg or proteins at 10 μg. Irrelevant mAb mRNA/LNPs (10 μg) were used as a negative control. At day 7 post-injection, mice were i.n. infected with SARS-CoV-2 BA.1 virus. (E) Viral titers in lung and nasal turbinate homogenates were quantified by TCID50 assay at day 4 post-infection. Data are shown as median ± IQR. Statistical significance was determined using a Kruskal-Wallis test followed by post-hoc Dunn's multiple comparisons test.

Pharmacokinetics and protective capacity of mRNA-delivered mAbs against SARS-CoV-2

We next compared the serum pharmacokinetics (PK) of mAbs delivered via ALC-0315 mRNA/LNPs versus recombinant proteins after i.v. administration (Figure 3A). Both mRNA and recombinant protein were administered at 1, 5, and 10 μg doses per animal and monitored over a 70-day sampling period. Peak serum concentrations of PDI204 were achieved at day 1 after administration of recombinant

Table 1. PK parameters for serum PDI204 delivered via mRNA/LNPs and protein

	Half-life (day)				
PDI204	Before day 7	After day 7	T_{max} (day)	C_{max} (μg/mL)	AUC_{last} (day*μg/mL)
mRNA_10μg	7.7 ± 1.2	1.8 ± 0.5	2.5 ± 1.0	3.4 ± 0.6	26.6 ± 7.2
mRNA_5μg	8.1 ± 3.1	2.3 ± 0.6	5.0	1.7 ± 0.5	11.6 ± 3.5
mRNA_1μg	3.2 ± 0.8	2.0 ± 0.9	2.6 ± 0.9	0.3 ± 0.1	1.9 ± 0.3
Protein_10μg	10.4 ± 4.0	12.7 ± 2.5	1.8 ± 1.1	2.2 ± 0.3	39.2 ± 5.1
Protein_5μg	9.7 ± 2.9	11.5 ± 2.7	1.0	1.0 ± 0.07	13.2 ± 0.9
Protein_1μg	6.4 ± 2.3	3.0 ± 1.2	1.0	0.08 ± 0.02	0.6 ± 0.2

protein, while for mRNA/LNPs, we generally observed that serum concentrations continued to increase out to days 3–5 (Figure 3B; Table 1). Both protein and mRNA displayed similar dose-response relationships; however, we observed marked differences in their persistence. While recombinant PDI204 proteins remained detectable up to day 70 when given at 5 or 10 μg doses, serum concentrations of mRNA-delivered mAbs rapidly declined from 7 days post-administration and were generally cleared by day 28 (Figure 3B). PK parameters are summarized in Table 1.

The Comirnaty formulation was developed for vaccine delivery and has been demonstrated to induce strong immune responses to the encoded antigen,[6] with the ionizable lipid ALC-0315 acting as a robust immune adjuvant.[20] We therefore assessed the induction of ADA (anti-PDI204) in mouse serum at days 1, 7, and 14 post-administration (Figure 3C). In contrast to mice administered a high dose of PDI204 protein (10 μg), all animals given mRNA/LNPs developed a significant antibody response against PDI204 over time. The findings suggest that the rapid reduction in serological titers of mRNA-delivered PDI204 seen from day 7 post-administration was likely attributed to ADA responses, with ADA induction likely enhanced by the immunogenic LNP delivery system and the species mismatch between the human IgG and the mouse host. Despite these ADA responses, neutralization assays showed that approximately 50% of PDI204 activity was retained in serum from day-28 mRNA/LNP-treated mice, indicating that residual antibody remained functionally active (Figure S1). This suggests that ADA did not fully block antiviral activity and may preferentially target non-antigen-binding regions of the antibody.

We next assessed the prophylactic efficacy of mRNA/LNP-delivered PDI204 against SARS-CoV-2 challenge in a transgenic human ACE2 mouse model (k18-hACE2).[14] At day 7 post i.v. injection of mAbs, the mice were intranasally infected with the SARS-CoV-2 Omicron BA.1 variant (strain SARS-CoV-2/Australia/NSW/RPAH-1933/2021) (Figure 3D). As the BA.1 strain causes limited pathology or weight loss in this model, we measured viral titers in the lungs and nasal turbinates at day 4 post-infection, corresponding to day 11 post-mAb administration (Figure 3E). Treatment with 10 μg PDI204 protein resulted in robust protection of the lungs, with no viral loads detected post-challenge (p = 0.0072). For animals receiving mRNA/LNPs, we observed greater animal-to-animal variability, but there was a dose-proportional reduction in viral loads associated with increasing treatment doses from 1 to 10 μg. Small and comparable amounts of virus were detected within the nasal turbinates of all challenged animals, likely reflective of the initial inoculum. Overall, the challenge study indicates that mRNA/LNP delivery of anti-viral mAbs can afford a degree of protection against SARS-CoV-2, although it was inferior to recombinant protein, likely due to the development of ADA over the extended 11-day challenge window.

Influenza mAbs delivered by mRNA/LNPs protect mice against lethal viral challenge

The influenza virus is a highly infectious respiratory pathogen that causes seasonal epidemics and periodic pandemics. We therefore explored the potential of mRNA/LNP delivery of a human anti-influenza mAb and assessed its protection against lethal influenza infection. HV-B10 was selected as a prototypic potent neutralizing human IgG1 mAb targeting the hemagglutinin (HA) on influenza pH1N1 viruses.[15] The HV-B10 mAb mRNA was also designed with HC and LC linked by a P2A motif.

The PK profile of HV-B10 delivered using ALC-0315 mRNA/LNPs was compared to recombinant protein as before (Figure 4A). Similar to PDI204, we found that administration of HV-B10 protein drove a rapid rise in serum concentration of mAb, followed by an extended decay period of more than 50 days for animals receiving 5 or 10 μg doses (Figures 4B and S2). In contrast, mRNA-delivered HV-B10 rapidly achieved high serum concentrations but was subject to rapid clearance from day 7 onward (Table 2). This again likely corresponded with the induction of anti-HV-B10 antibodies, which were readily evident in mice at days 7 and 14 post-mRNA/LNP administration (Figure 4C). Serum concentrations of HV-B10 after mRNA delivery peaked at higher levels than corresponding doses of recombinant protein and at levels higher than those seen with PDI204, suggesting that the specific characteristics of each mRNA construct can influence overall delivery efficiency *in vivo*.

The protective capacity of HV-B10 delivered as mRNA/LNPs or protein was assessed in animals intranasally challenged with a lethal dose (100 pfu) of A/California/04/2009 virus 1 day after mAb infusion (Figure 4D).[21] Mice receiving HV-B10 as recombinant protein showed a degree of protection at a 10 μg dose (approximately equivalent to 0.5 mg/kg in a ∼20 g animal), with delayed weight loss and 60% survival (Figure 4E). Doses of 1 and 5 μg of HV-B10 protein were not protective relative to controls. In contrast, all mice receiving HV-B10 mRNA at 1, 5, or 10 μg doses were protected from viral challenge, with a dose-associated reduction in weight loss observed. To assess how the timing of mRNA/LNP administration affects protective efficacy, mice were i.v. injected with 5 μg HV-B10 mRNA/LNPs either 14, 7, or 1 day before viral challenge (Figure S3A). Consistent with our earlier results (Figures 4B and 4C), longer intervals between injection and infection were associated with lower serum HV-B10 concentrations and higher ADA titers (Figures S3B

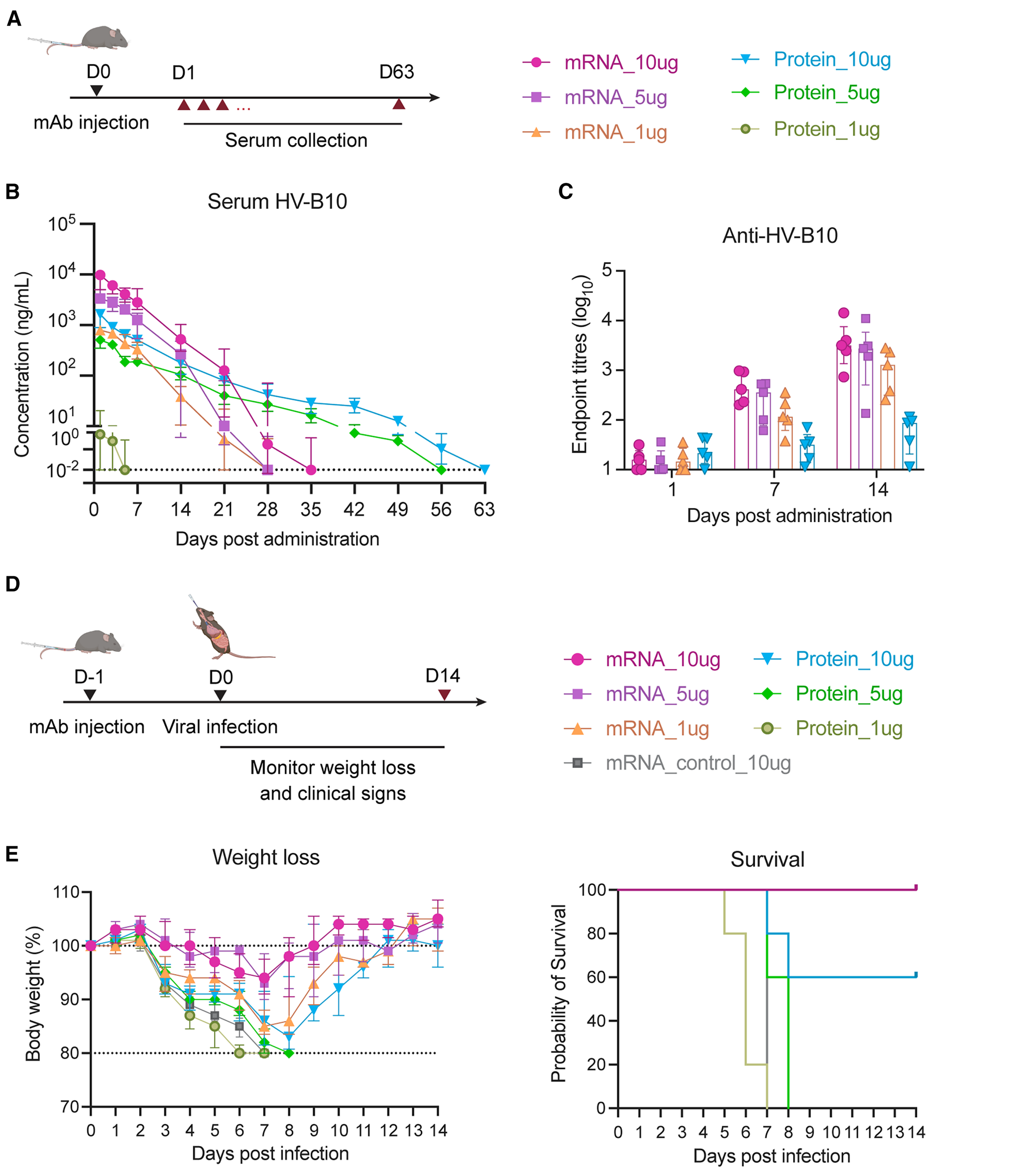

Figure 4. PK profile and protection efficacy of HV-B10 mRNA/LNPs against influenza infection

(A) HV-B10 mRNA/LNPs or proteins at 1, 5, or 10 μg were i.v. injected into C57BL/6 mice (*n* = 5). Sera from injected mice were collected at 1, 3, 5, 7, 14, 21, 28, 35, 42, 49, 56, and 63 days post-injection. (B) HV-B10 concentrations in sera over time were calculated using ELISA against A/California/04/2009 HA protein. (C) Anti-HV-B10 antibody titers in mouse serum at days 1, 7, and 14 post-injection were measured by ELISA. (D) At 24 h post mAb injection, mice were i.n. infected with 100 pfu of A/California/04/2009 virus. Irrelevant mAb mRNA/LNPs (10 μg) were used as a negative control. (E) Changes in body weight (left) and survival rates (right) of injected mice over 14 days post-viral challenge. Data are shown as median ± IQR.

Table 2. PK parameters for serum HV-B10 delivered via mRNA/LNPs and protein

HV-B10	Half-life (day) Before day 7	After day 7	T_{max} (day)	C_{max} (μg/mL)	AUC_{last} (day*μg/mL)
mRNA_10μg	5.3 ± 2.4	2.4 ± 1.1	1.4 ± 0.9	8.7 ± 2.9	49.5 ± 19.1
mRNA_5μg	3.9 ± 1.5	1.8 ± 0.6	1.0	3.8 ± 1.2	21.3 ± 7.6
mRNA_1μg	4.6 ± 1.5	2.4 ± 1.0	1.8 ± 1.1	0.8 ± 0.1	5.0 ± 1.0
Protein_10μg	6.7 ± 3.2	6.5 ± 0.7	1.4 ± 0.9	1.5 ± 0.5	9.4 ± 0.9
Protein_5μg	5.3 ± 1.2	7.2 ± 0.8	1.4 ± 0.9	0.5 ± 0.1	4.0 ± 0.8
Protein_1μg	1.3 ± 0.6	–	1.0	0.02 ± 0.01	0.03 ± 0.02

and S3C). These changes corresponded with increased body-weight loss, although all mice remained protected (Figure S3D). Overall, HV-B10 was efficiently delivered by mRNA-LNPs, which afforded superior protection against lethal influenza virus challenge compared to equivalent recombinant protein doses, although the level of protection depended on both the administered dose and the timing of viral challenge.

DISCUSSION

While vaccination represents a key tool for population-level protection from viral infection, antiviral therapeutics such as mAbs are important for supplemental protection of vulnerable subpopulations (e.g., immunocompromised or elderly individuals). mRNA/LNP technology afforded rapid design, development, and deployment of vaccines during the recent COVID-19 pandemic, which could be similarly applied for the delivery of mAb-based antivirals. Here, we tested mRNA/LNP delivery platforms for two antiviral mAbs in pre-clinical models of infection. In line with previous reports in pre-clinical[9,10] and clinical studies,[11] we found that co-formulation of HC and LC mRNA cargoes could efficiently drive mAb expression *in vitro* and *in vivo*, although the use of a single mRNA with HC and LC linked by P2A peptides was superior *in vivo* and potentially simpler to manufacture and formulate. The lower mAb expression observed with separate HC and LC mRNA/LNPs may be due to differential delivery of the two chains to different cells, which may impact IgG expression or assembly.

The lipid components selected for LNP formulation have shown marked impacts on the efficiency of mRNA delivery, trafficking, and immunostimulatory activity.[22] A limited number of LNP formulations have advanced to human clinical use, although compositions with ionizable lipids ALC-0315 (BioNTech/Pfizer), SM-102 (Moderna), and Dlin-MC3-DMA (MC3; Onpattro/Alnylam Pharmaceuticals) have been extensively studied. We found that Comirnaty-like formulations could drive efficient mAb accumulation *in vivo* after i.v. and i.m but not i.n. administration. In contrast, MC3 formulations with the permanent cationic lipid 1,2-dioleoyl-3-trimethylammonium-propane (DOTAP) included, previously reported to enhance delivery to lung tissues,[18,19] were demonstrably inferior and failed to robustly induce mAb expression in treated mice. A prior study achieved detectable mAb levels in the lung following i.v. administration using smaller (∼90 nm) and more positively charged (+25 mV) LNPs; however, mAb concentrations in BALF remained ∼100-fold lower than serum levels obtained with a conventional formulation.[19] Together, these findings highlight the influence of LNP physicochemical properties and administration route on local antibody bioavailability.

The ionizable lipid component (ALC-0315) used in vaccine platforms such as the Comirnaty COVID-19 vaccine has reported adjuvant properties.[20] In this study, we found that mRNA-LNP delivery was associated with rapid ADA elicitation in the context of delivering cross-species human mAbs into mice, resulting in reduced *in vivo* half-life and rapid serum clearance relative to recombinant protein controls, consistent with other reports of rapid clearance of human mAbs after mRNA/LNP injection in mice.[23] In contrast, injection of recombinant mAbs alone did not drive significant ADA, thus it remains possible that the adjuvant properties of LNPs may contribute to ADA induction. However, we note that in species-matched contexts (i.e., delivery of human mAbs to human subjects), ADA elicitation is likely to be minimal.

Here, ADA and accelerated clearance are likely to have significantly affected the protective capacity of these mAbs during pathogenic challenge. In the context of SARS-CoV-2, when animals were challenged relatively late (11 days) after mRNA/LNP administration, protection was inferior to that in animals given PDI204 recombinant protein, which displayed sterilizing immunity in the lungs. In contrast, when animals were challenged with influenza only 1 day post-mRNA/LNP administration, protection from mRNA-expressed HV-B10 was high and potentially more robust than that observed with recombinant protein controls. Although immune responses to mRNA-encoded mAbs will be heavily diminished when antibodies from matched species are used, future advances in LNP formulation design to allow a better balance between efficient mAb expression and minimal immunogenicity, as well as the use of self-amplifying RNA to achieve effective expression at lower doses,[24] are likely to be critical for the clinical development of LNP-based mAb delivery strategies. Similar ADA responses have also been observed with other gene-based antibody delivery platforms, including DNA and viral vectors (e.g., AAV), particularly when antibodies from a different species are expressed, and these effects can be mitigated by transient immunodepletion or by using immunodeficient mouse models (e.g., Rag1 knockout strains).[25,26]

Overall, this study provides a comprehensive assessment of mRNA/LNP-encoded mAb delivery, comparing multiple mAb construct designs, LNP formulations, administration routes, and antiviral targets. We demonstrate that mRNA/LNP delivery of neutralizing mAbs is a tractable pathway to protection against respiratory viruses such as SARS-CoV-2 and influenza, achieving serum antibody concentrations comparable to recombinant protein administration. Our findings also highlight key determinants of successful delivery of mAbs using mRNA/LNPs, including formulation properties, route

of administration, and the impact of ADA when expressing antibodies from a different species, likely reflecting a model-specific limitation that will be critical to clarify for future translation. Further work is required to identify LNP formulations best suited for maximizing the accumulation, retention, and potentially mucosal localization of antiviral mAbs. Nevertheless, the rapid manufacturing and clinical development pathways for mRNA/LNP technologies make them particularly well suited to strengthen pandemic preparedness, with efficient and cost-effective delivery of antiviral antibodies or biologics constituting a readily exploitable pathway to better protect human populations.

MATERIALS AND METHODS

Animal details and ethics statement

C57BL/6 and k18-hACE2 mice were bred at Australian Bio Resources (ABR) or Ozgene (WA, Australia) and housed at the Peter Doherty Institute's Bioresources Facility (BRF). All procedures involving animals and live SARS-CoV-2 were conducted in an Office of the Gene Technology Regulator (OCTR)-approved Physical Containment Level 3 (PC3) facility at the Center for AgriBiosciences (AgriBio). All animal studies and related experimental procedures were approved by the University of Melbourne Animal Ethics Committee (nos. 24909 and 22954). Six to 12-week-old female mice (n = 5 per group) were used for all studies.

Production of human mAbs

SARS-CoV-2 spike- and influenza HA-specific B cells were sorted from cryopreserved human peripheral blood mononuclear cells (PBMCs), and single B cell receptors were sequenced, cloned, and expressed as recombinant mAbs as described previously.[14,15] Briefly, recombined HC (V-D-J) and LC (V-J) immunoglobulin sequences were synthesized (Genscript) and cloned into human IgG1 expression vectors. The expression plasmids were then transfected into Expi293F cells using ExpiFectamine transfection reagents (Thermo Fisher Scientific). The recombinant human IgG1 mAbs were purified from culture supernatants using Protein A affinity chromatography.

mAb mRNA design and synthesis

All mRNAs were synthesized at the BASE mRNA facility (The University of Queensland), according to the method published previously.[27] Briefly, a synthetic DNA encoding either the HC, LC, or both HC and LC linked by a P2A peptide (HC-P2A-LC) of the human IgG1 mAbs was obtained from Integrated DNA Technologies (Singapore). The DNA was then PCR amplified and used as a template for *in vitro* transcription of mRNA using T7 RNA polymerase (New England Biolab), with all uridine residues substituted by N1-methylpseudouridine (BOC Sciences). The mRNA products were purified using a Monarch RNA Cleanup Kit (NEB), eluted in 1 mM sodium citrate, and sterile-filtered with a 0.22-μm syringe filter.

Preparation of mAb mRNA/LNPs

mAb mRNA/LNPs were prepared using the microfluidic mixing method.[17,21] Briefly, lipids (DC Chemicals), including ionizable lipids (ALC-0315, Dlin-MC3-DMA), cationic lipid (DOTAP), helper lipid (1,2-distearoyl-sn-glycero-3-phosphocholine [DSPC]), cholesterol, and polyethylene glycol (PEG)-lipids (ALC-0159, DMG-PEG2000) at specific molecular ratios, were dissolved in ethanol where applicable. The mRNAs encoding HC and LC at 1:1 molar ratio admix, or HC-P2A-LC, were diluted in acetate buffer (pH 4.0). The two solutions were injected into a microfluidic Ignite cartridge using a NanoAssemblr Ignite system (Precision NanoSystems) at an aqueous-to-ethanol ratio of 3:1 (vol./vol.), a total flow rate of 8 mL min^{-1}, and a flow rate ratio of 3:1. The formulations were subsequently purified by dialysis against 10% sucrose in Tris-buffered saline (TBS) buffer (Sigma-Aldrich) over 18–20 h at room temperature. Afterward, the mRNA/LNPs were filtered using a Durapore 0.45-μm polyvinylidene fluoride (PVDF) membrane (Merck) before being stored at −80°C until use.

Characterization of mAb mRNA/LNPs

DLS: The dynamic diameter and zeta potential of mRNA LNPs (50–100 μg mL^{-1} in PBS) were measured by dynamic light scattering (DLS) using a Malvern Zetasizer Nano Series.

EE: The total amount of mRNA and unencapsulated mRNA in LNPs (with or without the addition of 1 μL of 10% Triton X-100 and continuous mixing at 300 rpm, 37°C for 8 min) were quantified using a Quant-iT RiboGreen RNA assay kit (Thermo Fisher Scientific) according to the manufacturer guidelines. The encapsulation efficiency (EE) of mRNA in LNPs was calculated using the following formula: EE% = (total mRNA – unencapsulated mRNA) $\div$ total mRNA $\times$ 100.

In vitro expression of mRNA-delivered mAbs

HEK293T cells were seeded in 6-well plates at $\sim 10^6$ cells per well and incubated overnight. The cells were then transfected with LNPs delivering HC and LC mRNA admix (1:1 molar ratio) or HC-P2A-LC mRNAs using Lipofectamine 3000 Transfection Reagents (Thermo Fisher Scientific) in Opti-MEM I Reduced Serum Medium (Thermo Fisher Scientific). Four hours later, the medium was replaced with DMEM (10% fetal calf serum (FCS), 1$\times$ penicillin-streptomycin-glutamine [PSG]). The supernatant was collected 48 h after transfection, and mAb expression was detected using ELISA as described below.

In vivo expression and PK/PD studies of mAb mRNA/LNPs

C57BL/6 mice were i.v. administered 200 μL of LNPs delivering HC and LC admix or HC-P2A-LC mRNAs into the tail veins. At 48 h post-injection, blood samples were collected by submandibular or cardiac bleeds, and serum was isolated by centrifugation. Expression of mRNA-delivered mAbs was confirmed by ELISA.

In PK studies, C57BL/6 mice were i.v. administered 200 μL of mAb mRNA/LNPs or mAb protein at 1, 5, or 10 μg in PBS. Time-series samples of mouse serum were collected. The mAb levels over time were quantified by ELISA and then used to estimate PK parameters for each dose event using non-compartmental analysis.

In pharmacodynamic (PD) studies, two LNP formulations (ALC-0315 and MC3/DOTAP) delivering PDI204 HC-P2A-LC mRNA at a dose of 5 μg were administered to C57BL/6 mice via three different routes (i.v., i.m., and i.n.). At 48 h post-administration, serum, BALF, and nasal wash samples were collected. For BALF and nasal wash collection, a small incision in the trachea was made to allow insertion of a 20G cannula. The lungs were washed three times with 600 μL of PBS via the cannula to harvest BALF samples. Nasal washes were taken by washing the nasal cavity three times with 200 μL PBS. The mAb expression in the samples was calculated using ELISA assays.

ELISA for mAb detection

mAb expression in cell supernatant, mouse serum, BALF, and nasal washes was detected by direct ELISA. 96-well MaxiSorp plates were coated with 100 μL of antigens (either SARS-CoV-2 spike or A/California/04/2009 HA protein) at 2 μg mL^{-1} in PBS overnight at 4°C. The plates were then blocked with 1% FCS for 1 h at room temperature. All serum samples were diluted 1:10 in blocking solution, while BALF samples and cell supernatants were used neat initially, followed by serial 4-fold dilutions. Nasal washes were used neat. The sample dilutions were added to the plates and incubated for 2 h at room temperature. The plates were then incubated with anti-human secondary antibodies conjugated withhorseradish peroxidase (HRP) at a 1:15,000 dilution in the blocking solution for 1 h at room temperature. The plates were developed with 80 μL of TMB for 8 min and the reaction was stopped with 50 μL of 0.16 M sulfuric acid. Absorbance was measured at 450 nm. mAb concentrations in the serum and BALF samples were quantified based on standard curves generated from mAb protein dilutions, while ELISA endpoint titers were calculated as the reciprocal of the sample dilution giving a signal 2× above background. For nasal washes, mAb levels were reported as optical density at 450 nm (OD_{450}).

ELISA to detect anti-mAb antibodies

Antibodies against mAbs in mouse serum at days 1, 7, and 14 following i.v. administration of mAb mRNA/LNPs and mAb proteins were calculated using a direct ELISA assay as described previously.[28] Here, the plates were coated with mAb proteins at 2 μg mL^{-1} in PBS overnight at 4°C. Serum samples were diluted 1:10 in blocking solution, followed by a 4-fold serial dilution. HRP-conjugated anti-mouse secondary antibody was used at a 1:15,000 dilution.

Microneutralization assay

Neutralization activity of PDI204 mAbs in mouse serum against the ancestral SARS-CoV-2 strain was performed as previously described.[29] Briefly, SARS-CoV-2 isolate CoV/Australia/VIC31/2020 was passaged in Vero cells and stored at −80°C.[30] Infectivity of virus stocks was determined by titration on HAT-24 cells (a clone of transduced HEK293T cells stably expressing human ACE2 and TMPRSS2).[31] In a 96-well flat-bottom plate, virus stocks were serially diluted 5-fold (1:5–1:78125) in DMEM with 5% FCS. Then, 60,000 freshly trypsinized HAT-24 cells per well were added and incubated at 37°C. After 24 h, 10 μL of alamarBlue Cell Viability Reagent (Thermo Fisher Scientific) was added to each well and incubated at 37°C for 1 h. The reaction was stopped with 1% SDS and read on a FLUOstar Omega plate reader (excitation wavelength 560 nm, emission wavelength 590 nm). The relative fluorescent units (RFU) measured were used to calculate % viability ("sample" ÷ "no virus control" × 100), which was then plotted as a sigmoidal dose-response curve on Graphpad Prism to obtain the virus dilution that induces 50% cell death (50% lethal infectious dose; LD50). Virus stocks were titrated in quintuplicate in three independent experiments to obtain mean LD50 values.

To determine serum neutralization activity, heat-inactivated mouse serum samples were diluted 2.5-fold (1:50–1:30517) in duplicate and incubated with SARS-CoV-2 virus at a final concentration of 2 × LD50 at 37°C for 1 h. Next, 60 000 freshly trypsinized HAT-24 cells in DMEM with 5% FCS were added and incubated at 37°C. "Cells only" and "Virus+Cells" controls were included to represent 0% and 100% infectivity, respectively. After 24 h, 10 μL of alamarBlue Cell Viability Reagent (Thermo Fisher Scientific) was added to each well and incubated at 37°C for 1 h. The reaction was then stopped with 1% SDS and read on a FLUOstar Omega plate reader. The RFU measured were used to calculate % neutralization using the following formula: ("Sample" - "Virus+Cells") ÷ ("Cells only" - "Virus+Cells") × 100. Half-maximal inhibitory concentration (IC50) values were determined using four-parameter non-linear regression in GraphPad Prism, with the curve fit constrained to have a minimum of 0% and a maximum of 100% neutralization.

To assess the effect of ADA on the neutralizing capacity of PDI204, serial dilutions of PDI204 (from 500 ng mL^{-1} to 0.23 ng mL^{-1}) were pre-incubated with pooled sera (1:20 dilution) from either naive mice or mice containing high levels of anti-PDI204 ADA (at day 28 post 10 μg mRNA delivery of PDI204). After 1 h, SARS-CoV-2 virus was added, and the neutralization assay was conducted as described above.

Prophylaxis studies of mAb mRNA/LNPs against SARS-CoV-2 infection in mice

Prophylactic efficacy of PDI204 mRNA/LNPs versus proteins was assessed in K18-hACE2 mice. Briefly, K18-hACE2 mice were i.v. injected with 200 μL of PDI204 mRNA/LNPs at 1, 5, or 10 μg, protein, or irrelevant mAb mRNA/LNPs at 10 μg. Seven days post-treatment, mice were lightly anesthetized with isoflurane and intranasally infected with 10^4 50% tissue culture infectious dose (TCID50) of SARS-CoV-2 Omicron BA.1 (strain SARS-CoV-2/Australia/NSW/RPAH-1933/2021) in 50 μL PBS. On day 4 post-infection, mice were humanely euthanized. Nasal turbinates and lungs were collected and homogenized using an Omni tissue homogenizer in 1 mL or 2 mL of PBS, respectively, containing 50 U/mL of penicillin, 50 μg/mL streptomycin, and 4 μg/mL amphotericin B (Gibco). Homogenates were clarified by centrifugation at 2,000 × g for 10 min before virus quantification by a $TCID_{50}$ assay. Briefly, clarified tissue homogenates were serially

diluted 1:10 on confluent monolayers of VeroE6 cells overexpressing TMPRSS2 in DMEM containing 50 U/mL penicillin, 50 μg/mL streptomycin, 2 mM glutamax (Gibco), and 1 μg/mL trypsin (Worthington Biochemical) in quadruplicate. Plates were incubated at 37°C with 5% CO_2 for 4 days, and cytopathic effects wre measured under a light microscope. $TCID_{50}$ was determined using the Reed-Muench method. Virus titers are expressed as mean $\log_{10}$ $TCID_{50}$/mL.

Prophylaxis studies of mAb mRNA/LNPs against influenza infection in mice

Prophylactic efficacy of HV-B10 mRNA/LNPs versus proteins was assessed in murine infection models as described previously.[15,21] C57BL/6 mice were i.v. injected with 200 μL of HV-B10 mRNA/LNPs or protein at 1, 5, or 10 μg, or with irrelevant mAb mRNA/LNPs as a control (10 μg) 24 h prior to viral infection.

For the challenge-timing experiment, mice were injected with 5 μg HV-B10 mRNA/LNPs at 14, 7, or 1 day before infection or with 5 μg irrelevant mAb mRNA/LNPs 1 day prior.

Mice were then intranasally infected with 50 μL of A/California/04/2009 virus at a lethal dose of 100 pfu in PBS. Animals were monitored for clinical signs and weight loss for 14 days post-infection. Mice were euthanized if they lost 20% of their initial body weight.

Statistical analysis

Data were presented as median ± interquartile range (IQR) (n = 5). Statistical analyses were performed using GraphPad Prism version 10. For comparisons involving multiple groups, a Kruskal-Wallis test followed by post-hoc Dunn's multiple comparisons test was used. p values less than 0.05 ($p < 0.05$) were considered significant for all statistical tests.

DATA AND CODE AVAILABILITY

Any data are available upon request from the corresponding author.

ACKNOWLEDGMENTS

Some portions of the figures were created with BioRender (BioRender.com). All mAb mRNA constructs used in this study were synthesized by the BASE mRNA Facility at the University of Queensland. The authors acknowledge the facilities and technical assistance of the Bioresources Facility (BRF) at the University of Melbourne and the Center for AgriBiosciences (AgriBio) at La Trobe University. This work was supported by Australian Medical Research Future Fund grants 2005544 and 2013870, Australian National Health and Medical Research Council Investigator grants (W.S.L., H.-X.T., and A.K.W.), and Therapeutic Innovation Australia Pipeline Accelerator Voucher Program (M.N.V.).

AUTHOR CONTRIBUTIONS

M.N.V. and A.K.W. designed the study. M.N.V., J.A.N., C.M.-K., and W.S.L. performed experiments. A.K. synthesized and purified protein mAbs. H.-X.T., K.S., and W.S.L. provided guidance and discussion on experimental design. M.N.V. and A.K.W. wrote the manuscript. All authors read and revised the manuscript.

DECLARATION OF INTERESTS

The authors declare no competing interests.

SUPPLEMENTAL INFORMATION

Supplemental information can be found online at https://doi.org/10.1016/j.omtn.2026.102873.

REFERENCES

1. Khoury, D.S., Cromer, D., Reynaldi, A., Schlub, T.E., Wheatley, A.K., Juno, J.A., Subbarao, K., Kent, S.J., Triccas, J.A., and Davenport, M.P. (2021). Neutralizing antibody levels are highly predictive of immune protection from symptomatic SARS-CoV-2 infection. Nat. Med. *27*, 1205–1211. https://doi.org/10.1038/s41591-021-01377-8.
2. Coudeville, L., Bailleux, F., Riche, B., Megas, F., Andre, P., and Ecochard, R. (2010). Relationship between haemagglutination-inhibiting antibody titres and clinical protection against influenza: development and application of a bayesian random-effects model. BMC Med. Res. Methodol. *10*, 18. https://doi.org/10.1186/1471-2288-10-18.
3. Gaudinski, M.R., Coates, E.E., Novik, L., Widge, A., Houser, K.V., Burch, E., Holman, L.A., Gordon, I.J., Chen, G.L., Carter, C., et al. (2019). Safety, tolerability, pharmacokinetics, and immunogenicity of the therapeutic monoclonal antibody mAb114 targeting Ebola virus glycoprotein (VRC 608): an open-label phase 1 study. Lancet *393*, 889–898. https://doi.org/10.1016/S0140-6736(19)30036-4.
4. Venkatesan, P. (2022). Nirsevimab: a promising therapy for RSV. Lancet Microbe *3*, e335. https://doi.org/10.1016/S2666-5247(22)00097-0.
5. Corti, D., Purcell, L.A., Snell, G., and Veesler, D. (2021). Tackling COVID-19 with neutralizing monoclonal antibodies. Cell *184*, 3086–3108. https://doi.org/10.1016/j.cell.2021.05.005.
6. Dagan, N., Barda, N., Kepten, E., Miron, O., Perchik, S., Katz, M.A., Hernán, M.A., Lipsitch, M., Reis, B., and Balicer, R.D. (2021). BNT162b2 mRNA Covid-19 Vaccine in a Nationwide Mass Vaccination Setting. N. Engl. J. Med. *384*, 1412–1423.
7. Doria-Rose, N., Suthar, M.S., Makowski, M., O'Connell, S., McDermott, A.B., Flach, B., Ledgerwood, J.E., Mascola, J.R., Graham, B.S., Lin, B.C., et al. (2021). Antibody Persistence through 6 Months after the Second Dose of mRNA-1273 Vaccine for Covid-19. N. Engl. J. Med. *384*, 2259–2261.
8. Van Hoecke, L., and Roose, K. (2019). How mRNA therapeutics are entering the monoclonal antibody field. J. Transl. Med. *17*, 54. https://doi.org/10.1186/s12967-019-1804-8.
9. Deng, Y.Q., Zhang, N.N., Zhang, Y.F., Zhong, X., Xu, S., Qiu, H.Y., Wang, T.C., Zhao, H., Zhou, C., Zu, S.L., et al. (2022). Lipid nanoparticle-encapsulated mRNA antibody provides long-term protection against SARS-CoV-2 in mice and hamsters. Cell Res. *32*, 375–382. https://doi.org/10.1038/s41422-022-00630-0.
10. Lee, S.-Y., Lee, Y., Oh, E.Y., Lee, J., Kim, J.-Y., Park, S.-I., Park, H.-J., Park, S.H., Choi, E.-J., Ha, D., et al. (2025). The therapeutic potential of mRNA-encoded SFTSV human monoclonal antibody encapsulated lipid nanoparticle *in vivo*. J. Control. Release *382*, 113735. https://doi.org/10.1016/j.jconrel.2025.113735.
11. August, A., Attarwala, H.Z., Himansu, S., Kalidindi, S., Lu, S., Pajon, R., Han, S., Lecerf, J.-M., Tomassini, J.E., Hard, M., et al. (2021). A phase 1 trial of lipid-encapsulated mRNA encoding a monoclonal antibody with neutralizing activity against Chikungunya virus. Nat. Med. *27*, 2224–2233. https://doi.org/10.1038/s41591-021-01573-6.
12. Cox, R.J., and Pathirana, R. (2024). Unravelling influenza correlates of protection: lessons from human A/H1N1 Challenge. mBio *15*, e00064-24. https://doi.org/10.1128/mbio.00064-24.
13. Paul, M.J., Hudda, M.T., Pallett, S., Groppelli, E., Boariu, E., Finardi, N.F., Wake, R., Sofat, N., Biddle, K., Koushesh, S., et al. (2025). Mucosal immune responses to SARS-CoV-2 infection and COVID-19 vaccination. Vaccine *56*, 127175. https://doi.org/10.1016/j.vaccine.2025.127175.
14. Wheatley, A.K., Pymm, P., Esterbauer, R., Dietrich, M.H., Lee, W.S., Drew, D., Kelly, H.G., Chan, L.J., Mordant, F.L., Black, K.A., et al. (2021). Landscape of human antibody recognition of the SARS-CoV-2 receptor binding domain. Cell Rep. *37*, 109822. https://doi.org/10.1016/J.CELREP.2021.109822.
15. Vanderven, H.A., Esterbauer, R., Jegaskanda, S., Tan, H.-X., Wheatley, A.K., and Kent, S.J. (2022). Poor protective potential of influenza nucleoprotein antibodies despite wide prevalence. Immunol. Cell Biol. *100*, 49–60. https://doi.org/10.1111/imcb.12508.

16. Chng, J., Wang, T., Nian, R., Lau, A., Hoi, K.M., Ho, S.C.L., Gagnon, P., Bi, X., and Yang, Y. (2015). Cleavage efficient 2A peptides for high level monoclonal antibody expression in CHO cells. mAbs *7*, 403–412. https://doi.org/10.1080/19420862.2015.1008351.

17. Vu, M.N., Pilapitiya, D., Kelly, A., Koutsakos, M., Kent, S.J., Juno, J.A., Tan, H.-X., and Wheatley, A.K. (2025). Deconvolution of cargo delivery and immunogenicity following intranasal delivery of mRNA lipid nanoparticle vaccines. Mol. Ther. Nucleic Acids *36*, 102547. https://doi.org/10.1016/j.omtn.2025.102547.

18. Cheng, Q., Wei, T., Farbiak, L., Johnson, L.T., Dilliard, S.A., and Siegwart, D.J. (2020). Selective organ targeting (SORT) nanoparticles for tissue-specific mRNA delivery and CRISPR–Cas gene editing. Nat. Nanotechnol. *15*, 313–320. https://doi.org/10.1038/s41565-020-0669-6.

19. Tai, W., Yang, K., Liu, Y., Li, R., Feng, S., Chai, B., Zhuang, X., Qi, S., Shi, H., Liu, Z., et al. (2023). A lung-selective delivery of mRNA encoding broadly neutralizing antibody against SARS-CoV-2 infection. Nat. Commun. *14*, 8042. https://doi.org/10.1038/s41467-023-43798-8.

20. Alameh, M.G., Tombácz, I., Bettini, E., Lederer, K., Sittplangkoon, C., Wilmore, J.R., Gaudette, B.T., Soliman, O.Y., Pine, M., Hicks, P., et al. (2021). Lipid nanoparticles enhance the efficacy of mRNA and protein subunit vaccines by inducing robust T follicular helper cell and humoral responses. Immunity *54*, 2877–2892.e7. https://doi.org/10.1016/j.immuni.2021.11.001.

21. Vu, M.N., Kelly, H.G., Tan, H.-X., Juno, J.A., Esterbauer, R., Davis, T.P., Truong, N.P., Wheatley, A.K., and Kent, S.J. (2021). Hemagglutinin Functionalized Liposomal Vaccines Enhance Germinal Center and Follicular Helper T Cell Immunity. Adv. Healthc. Mater. *10*, 2002142. https://doi.org/10.1002/adhm.202002142.

22. Hou, X., Zaks, T., Langer, R., and Dong, Y. (2021). Lipid nanoparticles for mRNA delivery. Nat. Rev. Mater. *6*, 1078–1094. https://doi.org/10.1038/s41578-021-00358-0.

23. Hazell, N.C., Reyna, R.A., Adam, A., Bonam, S.R., Bei, J., Kumar, N., Nugyen, T., Plante, J.A., Walker, D.H., Wang, T., et al. (2025). mRNA-delivered neutralizing antibodies confer protection against SARS-CoV-2 variant in the lower and upper respiratory tract. Preprint at bioRxiv. https://doi.org/10.1101/2025.04.28.650951.

24. McKay, P.F., Hu, K., Blakney, A.K., Samnuan, K., Brown, J.C., Penn, R., Zhou, J., Bouton, C.R., Rogers, P., Polra, K., et al. (2020). Self-amplifying RNA SARS-CoV-2 lipid nanoparticle vaccine candidate induces high neutralizing antibody titers in mice. Nat. Commun. *11*, 3523. https://doi.org/10.1038/s41467-020-17409-9.

25. Wise, M.C., Xu, Z., Tello-Ruiz, E., Beck, C., Trautz, A., Patel, A., Elliott, S.T., Chokkalingam, N., Kim, S., Kerkau, M.G., et al. (2020). In vivo delivery of synthetic DNA–encoded antibodies induces broad HIV-1–neutralizing activity. J. Clin. Investig. *130*, 827–837. https://doi.org/10.1172/JCI132779.

26. Lin, A., and Balazs, A.B. (2018). Adeno-associated virus gene delivery of broadly neutralizing antibodies as prevention and therapy against HIV-1. Retrovirology *15*, 66. https://doi.org/10.1186/s12977-018-0449-7.

27. Leighton, L.J., Chaudhary, N., Tompkins, H.T., Kulkarni, A., Carrodus, N.L., Budzinska, M.A., Lakshman Das, S., Cheetham, S.W., and Mercer, T.R. (2025). The design, manufacture and LNP formulation of mRNA for research use. Nat. Protoc. *20*, 3552–3581. https://doi.org/10.1038/s41596-025-01174-4.

28. Rane, S.S., Dearman, R.J., Kimber, I., and Derrick, J.P. (2022). Adaptation of an ELISA assay for detection of IgG2a responses against therapeutic monoclonal antibodies in a mouse immunization model. J. Immunotoxicol. *19*, 1–7. https://doi.org/10.1080/1547691X.2021.2020937.

29. Vu, M.N., Pilkington, E.H., Lee, W.S., Tan, H.-X., Davis, T.P., Truong, N.P., Kent, S.J., and Wheatley, A.K. (2023). Engineered Ferritin Nanoparticle Vaccines Enable Rapid Screening of Antibody Functionalization to Boost Immune Responses. Adv. Healthc. Mater. *12*, 2202595. https://doi.org/10.1002/ADHM.202202595.

30. Caly, L., Druce, J., Roberts, J., Bond, K., Tran, T., Kostecki, R., Yoga, Y., Naughton, W., Taiaroa, G., Seemann, T., et al. (2020). Isolation and rapid sharing of the 2019 novel coronavirus (SARS-CoV-2) from the first patient diagnosed with COVID-19 in Australia. Med. J. Aust. *212*, 459–462. https://doi.org/10.5694/mja2.50569.

31. Tea, F., Ospina Stella, A., Aggarwal, A., Ross Darley, D., Pilli, D., Vitale, D., Merheb, V., Lee, F.X.Z., Cunningham, P., Walker, G.J., et al. (2021). SARS-CoV-2 neutralizing antibodies: Longevity, breadth, and evasion by emerging viral variants. PLoS Med. *18*, e1003656. https://doi.org/10.1371/JOURNAL.PMED.1003656.

OMTN, Volume *37*

Supplemental information

Delivery of monoclonal antibodies using mRNA lipid nanoparticles confers protection against SARS-CoV-2 and influenza

Mai N. Vu, Jessica A. Neil, Charley Mackenzie-Kludas, Andrew Kelly, Hyon-Xhi Tan, Kanta Subbarao, Wen Shi Lee, and Adam K. Wheatley

Table S1: Formulation of mAb mRNA/LNPs

Formulation	Lipid components*	Lipid ratio*	N:P ratio
ALC-0315	ALC-0315, DSPC, cholesterol, ALC-0159	46.3/9.4/42.7/1.6	1:5
MC3/DOTAP	Dlin-MC3-DMA, DSPC, cholesterol, DSPE-PEG2000, DOTAP	25.0/5.0/19.2/0.8/50.0	1:6

*Ionizable lipid, helper lipid, cholesterol, PEG-lipid, cationic lipid

Table S2: Characterisation of mRNA/LNPs

Formulation	mRNA	Diameter (nm)	PdI	Zeta potential (mV)	Encapsulation efficiency (%)
ALC-0315	HC/LC admix	62.3 ± 1.5	0.102	- 8.31	89.3
	HC-P2A-LC	64.1 ± 1.3	0.116	- 8.09	90.6
MC3-DOTAP	HC-P2A-LC	121.7 ± 4.2	0.146	+ 15.2	96.5

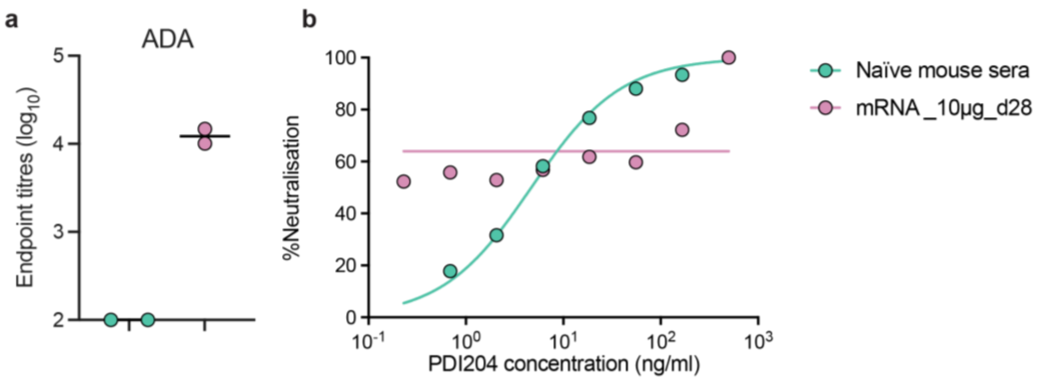

Figure S1: Effects of anti-PDI204 ADA on neutralisation activity of PDI204. Sera from naïve mice and from mice intravenously injected with 10 µg PDI204 mRNA/LNPs were collected at day 28 (the latest time point at which PDI204 was detectable) and pooled. (a) Anti-PDI204 ADA titres in pooled sera. (b) Neutralisation activity of PDI204 in the presence of pooled mouse sera against ancestral SARS-CoV-2 measured by a microneutralisation assay.

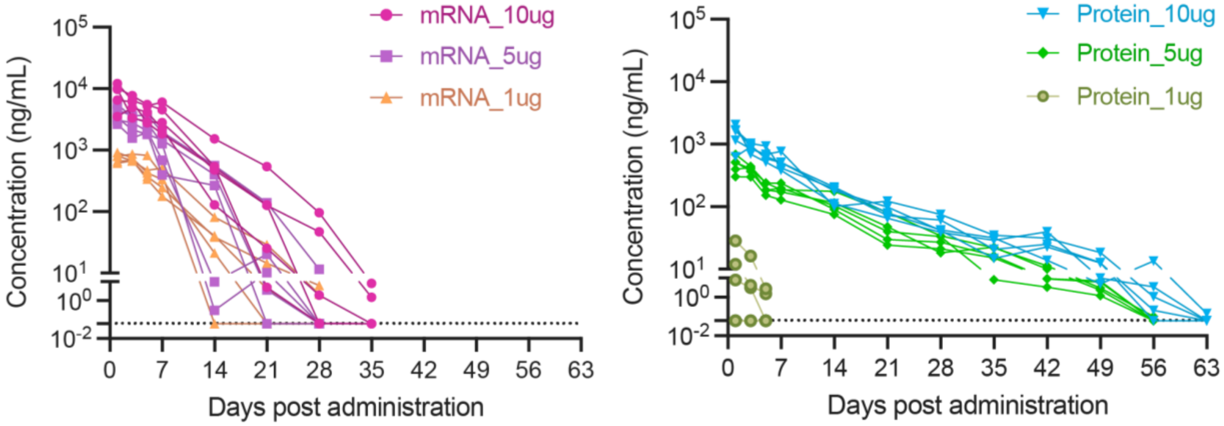

Figure S2: **PK profile of individual animals administered with HV-B10 mRNA/LNPs and protein.** HV-B10 mRNA/LNPs or proteins at 1, 5, or 10 µg were i.v. injected into C57BL/6 mice (n = 5). Sera from injected mice were collected at 1, 3, 5, 7, 14, 21, 28, 35, 42, 49, 56, and 63 days post injection. HV-B10 concentrations in the sera over time were calculated using ELISA against A/California/04/2009 HA protein (related to Figure 4b).

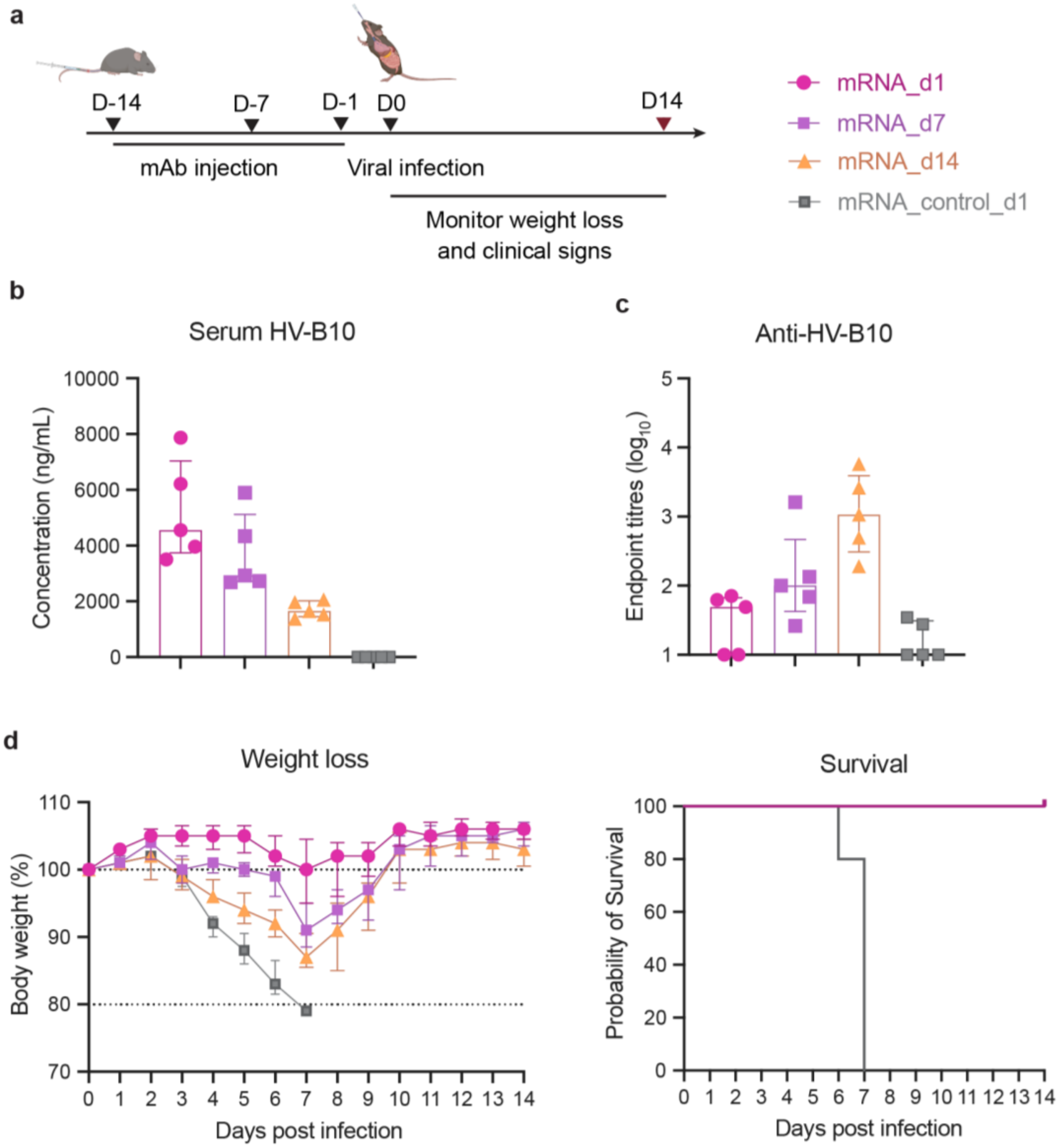

Figure S3: Timing of administration affects the protection efficacy of HV-B10 mRNA/LNPs against influenza infection. a). HV-B10 mRNA/LNPs at 5 µg were i.v. injected into C57BL/6 mice (n = 5) at day -14, -7, and -1 and an irrelevant mAb mRNA/LNPs at 5 µg were i.v. injected at day -1 as a negative control. At day 0, the mice were i.n. infected with 100 pfu of A/California/04/2009 virus. b). HV-B10 concentrations in the sera collected prior to infection were calculated using ELISA against A/California/04/2009 HA protein. c). Anti-HV-B10 anti-drug antibody titres in the mouse serum were measured by an ELISA assay. d). Changes in body weight (left) and survival rates (right) of the mice in 14 days post viral challenge. Data are shown as median ± IQR.
